# Aberrant Promoter Methylation of the Tumour Suppressor RASSF10 and Its Growth Inhibitory Function in Breast Cancer

**DOI:** 10.3390/cancers8030026

**Published:** 2016-02-25

**Authors:** Antje M. Richter, Sara K. Walesch, Reinhard H. Dammann

**Affiliations:** Institute for Genetics, University of Giessen, Giessen 35392, Germany; sara.walesch@gen.bio.uni-giessen.de (S.K.W.); reinhard.dammann@gen.bio.uni-giessen.de (R.H.D.)

**Keywords:** breast cancer, tumour suppressor, DNA methylation, epigenetics, RASSF

## Abstract

Breast cancer is the most common cancer in women, with 1.7 million new cases each year. As early diagnosis and prognosis are crucial factors in cancer treatment, we investigated potential DNA methylation biomarkers of the tumour suppressor family Ras-association domain family (RASSF). Promoter hypermethylation of tumour suppressors leads to their inactivation and thereby promotes cancer development and progression. In this study we analysed the tumour suppressors RASSF1A and RASSF10. Our study shows that RASSF10 is expressed in normal breast but inactivated by methylation in breast cancer. We observed a significant inactivating promoter methylation of RASSF10 in primary breast tumours. RASSF10 is inactivated in 63% of primary breast cancer samples but only 4% of normal control breast tissue is methylated (*p* < 0.005). RASSF1A also shows high promoter methylation levels in breast cancer of 56% *vs.* 8% of normal tissue (*p* < 0.005). Interestingly more than 80% of breast cancer samples harboured a hypermethylation of RASSF10 and/or RASSF1A promoter. Matching samples exhibited a strong tumour specific promoter methylation of RASSF10 in comparison to the normal control breast tissue. Demethylation treatment of breast cancer cell lines MCF7 and T47D reversed RASSF10 promoter hypermethylation and re-established RASSF10 expression. In addition, we could show the growth inhibitory potential of RASSF10 in breast cancer cell lines MCF7 and T47D upon exogenous expression of RASSF10 by colony formation. We could further show, that RASSF10 induced apoptotic changes in MCF7 and T47D cells, which was verified by a significant increase in the apoptotic sub G1 fraction by 50% using flow cytometry for MCF7 cells. In summary, our study shows the breast tumour specific inactivation of RASSF10 and RASSF1A due to DNA methylation of their CpG island promoters. Furthermore RASSF10 was characterised by the ability to block growth of breast cancer cell lines by apoptosis induction.

## 1. Introduction

Breast cancer is the most common cancer in women. It is the second most common overall cancer type, accounting for almost 1.7 million new cases among the 14 million total new cancer cases worldwide in 2012 [1]. It ranks fifth as cause of death from cancer overall, due to its favourable prognosis [1]. Breast cancer is histologically classified into non-invasive (*in situ*) and invasive (infiltrating) carcinomas and breast cancer metastasis occurs mainly into adjacent lymph nodes [2]. Treatment possibilities span from surgical removal, chemotherapy, radiotherapy, endocrine hormone therapy to targeted therapy [3]. 70% of breast tumours are positive for the estrogen receptor, which can be targeted by endocrine therapy [4]. 20%–25% of breast cancers have an amplified HER2/neu growth factor and can be targeted by anti HER2 antibody [5]. Mutations in the BRCA1/2 tumour suppressors are linked to familial predisposition to breast cancer [6]. However not only mutations inactivate tumour suppressors, but also promoter hypermethylation at CpG islands inactivates expression of the according transcripts [7]. Aberrant methylation patterns were reported for e.g. the BRCA1 promoter [8] and the tumour suppressor *RASSF1A* in breast cancer [9]. *RASSF1A* was reviewed as a candidate DNA methylation marker not only in breast cancer [10]. The tumour suppressor family Ras-Association domain Family (RASSF) consists of 10 members as well as various isoforms and is further divided into two subfamilies [11,12]. The *C*-terminal members RASSF1 to RASSF6 contain the Ras-association domain (RA) at the *C*-terminus and an additional SARAH protein interaction domain. The *N*-terminal members RASSF7 to RASSF10 harbour the Ras-association domain at the *N*-terminus and contain coiled coils for protein interaction [12]. The RA domain can be found in proteins interacting with RAS members [13] and the SARAH domain is a typical representative of protein interaction within the Hippo signalling pathway [14]. The Hippo pathway is a crucial regulator of organ size [15]. Besides interaction domains, no catalytically active domains are found in the RASSF member [11,12]. In this study we analysed the most prominent members of the *C*- and *N*-terminal RASSF family: RASSF1A and RASSF10. Both RASSFs contain characteristic CpG Islands within their promoter region (UCSC genome browser, CpG plot http://www.ebi.ac.uk/Tools/seqstats/emboss_cpgplot/) and are prone to epigenetic silencing by DNA methylation in cancer [11,16]. RASSF1A is established as an epigenetic inactivated tumour suppressor in breast cancer [10] and functions in microtubule stabilisation, cell cycle inhibition and apoptosis induction [11]. It was first described in 2000 by Dammann *et al.* [17]. This study and in our recent work we focussed on *RASSF10*, that exhibits CpG island hypermethylation in various cancer types [16,18,19,20,21,22,23,24]. To date however less information exists on RASSF10’s tumour suppressive function. So far it is known that RASSF10 blocks tumour cell growth *in vitro* and in xenograft [16,18,25]. Our own work showed that activation of cAMP signalling upregulated *RASSF10* expression, which linked RASSF10 function to extracellular stimuli [16]. In this present study we mainly focussed on the contribution of *RASSF10* promoter methylation in breast cancer. Additionally we studied demethylation and reversal of *RASSF10* expression as well as RASSF10’s tumour suppressive function in breast cancer.

## 2. Results

**Figure 1:**
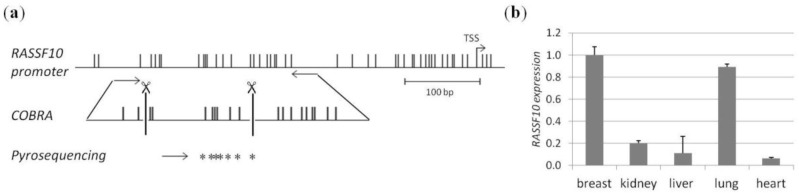
*RASSF10* promoter structure and normal expression levels (**a**) The *RASSF10* promoter with its CpG island structure. Black vertical lines represent single CpGs and restriction enzyme *Taq*I recognition sites are marked. Bent arrow indicates transcriptional start site (TSS). Horizontal arrows mark PCR products. PCR product size was 241 bp for COBRA methylation analysis. Restriction site positions for COBRA are shown. Pyrosequencing covers seven CpGs (asterisks) within the COBRA analysed region. 100 bp standard is shown; (**b**) *RASSF10* expression is shown relative to *b-Actin* expression in human breast, kidney, liver, lung and heart RNA samples of healthy donors after reverse transcriptase PCR and quantitative PCR.

In this study we analysed the promoter inactivation by DNA methylation of *RASSF10* in breast cancer. RASSF10 is a member of the well known tumour suppressor family named RASSF. *RASSF10* contains a more than 2 kb large CpG islands in its promoter region as analysed by CpG plot and UCSC Genome browser (Figure 1a).

Within this region COBRA methylation analysis primers were placed. We used the COBRA technique for methylation analysis for breast cancer cell lines, primary breast samples and control tissues. Our findings were quantified using pyrosequencing covering seven CpGs within the COBRA region. At first *RASSF10* expression was determined in normal samples from healthy donors (Figure 1b). The normal RNA panel was obtained from Agilent (Waldbronn, Germany). We could show that *RASSF10* expression is highest in breast *vs.* kidney, liver, lung and heart. However expression in the lung is 10% below expression in the breast. We were unable to study RASSF10 expression on protein level due to the lack of an appropriate antibody.

**Figure 2:**
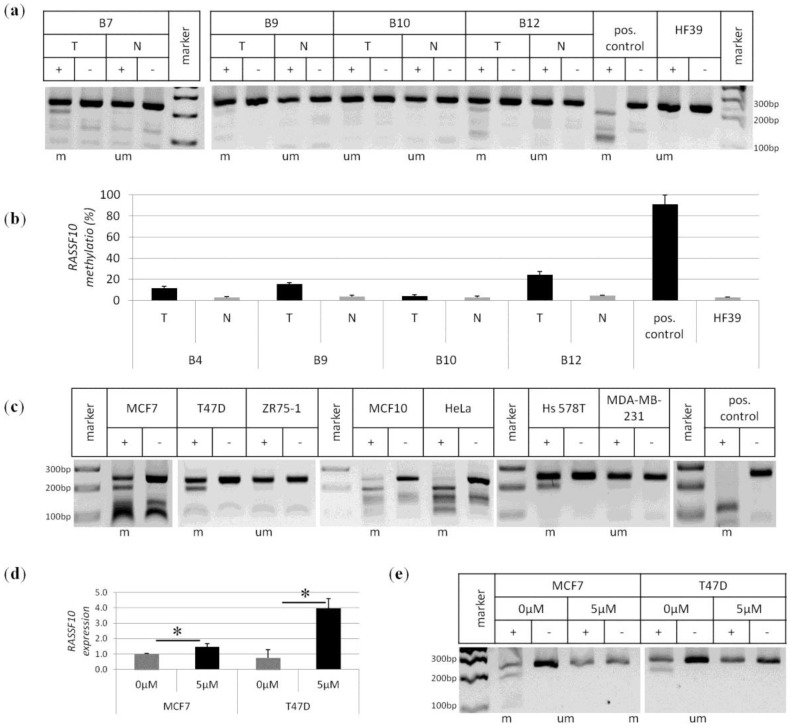
*RASSF10* promoter methylation in primary breast tumours and breast cancer cell lines. (**a**) *RASSF10* COBRA promoter methylation analysis is shown exemplarily for samples of primary breast cancer (T) and normal matching control tissues (N). Positive control shows full methylation of *RASSF10* promoter *vs.* human fibroblasts HF39 as negative control. PCR products for the *RASSF10* promoter from bisulfite treated DNA were digested (+) with *Taq*I or mock digested (−). According digestion products were separated on 2% TBE gel together with 100 bp marker. Methylated samples (m) and unmethylated samples (um) are indicated; (**b**) *RASSF10* promoter methylation was quantified by pyrosequencing for breast tumour samples (T) in comparison to normal matching control samples (N). As methylation controls HeLa (positive control) and human fibroblasts (HF39, negative control) were used. Mean of seven CpGs is shown; (**c**) *RASSF10* promoter methylation of cancer cell lines is shown by COBRA. Breast cancer cell lines MCF7, T74D, MCF10 and Hs578T are methylated to a varying degree, but ZR75-1 and MDA-MB-231 were unmethylated for *RASSF10*. As positive control cervix carcinoma cell line HeLa and ivm DNA (pos. control) were used; (**d** and **e**) Treatment of breast cancer cell lines with demethylating agent 5-Aza-2′-deoxycytidin reverses *RASSF10* expression repression due to DNA promoter demethylation. Cell lines MCF7 and T47D were treated with 5 μM Aza or mock on four consecutive days followed by DNA and RNA isolation. RNA was reversely transcribed and *RASSF10* reexpression was normalised to *b-Actin.* Significant reexpression (asterisks) was determined by two-tailed *T*-Test for MCF7 *p* < 0.05 and T47D *p* < 0.005 (**d**); Isolated DNA was bisulfite treated and *RASSF10* promoter demethylation was analysed by COBRA (**e**).

Next we analysed breast tumour and control samples by COBRA methylation analysis for the *RASSF10* promoter. Results are shown exemplarily (Figure 2a). DNA from breast samples was bisulfite treated and a specific region within the *RASSF10* CpG island promoter was amplified by PCR. Next the PCR products of 241 bp were digested using *Taq*I enzyme that distinguishes between originally methylated or unmethylated promoter regions. Full *RASSF10* methylation is shown by digestion products of 50 bp, 91 bp and 100 bp of the 241 bp PCR product. Partial promoter methylation gives different combination of fragment sizes. Promoter methylation can be observed in the case of sample B7 tumour, B9 tumour and B12 tumour. A positive control cell line and human fibroblasts as a negative control are shown. Using pyrosequencing we quantified promoter methylation of *RASSF10* for a set of samples (Figure 2b) and observed a clear tumour specific *RASSF10* promoter methylation *versus* normal matching tissue. Pyrosequencing of the *RASSF10* promoter in these breast samples confirmed that even lower methylation levels (e.g. sample B9, B12) corresponded to the COBRA results (Figure 2a). Pyrosequencing results are shown as mean methylation of seven CpGs (Figure 1a). Human breast cancer cell lines are strongly methylated for the *RASSF10* promoter (Figure 2c), and methylation levels vary. MCF7, T74D and Hs578T are partially methylated, ZR75-1 and MDA-MB-231 are unmethylated and MCF10 is fully methylated. The cervix carcinoma cell line HeLa, due to its strong *RASSF10* methylation, and an *in vitro* methylated DNA were used as positive controls [16]. Breast cancer cell lines MCF7 and T47D were chosen for further analysis due to the present *RASSF10* promoter methylation. These cell lines were treated with 5-Aza-2′deoxycytidine (Aza) a DNA methyltransferase inhibitor, which blocks maintenance methylation through DNMTs. *RASSF10* promoter methylation was decreased upon treatment with 5 μM of Aza as shown by COBRA analysis (Figure 2e). This observation was accompanied by a significant reexpression of *RASSF10* RNA levels and was strongest for the T47D cancer cell line (Figure 2d).

**Figure 3:**
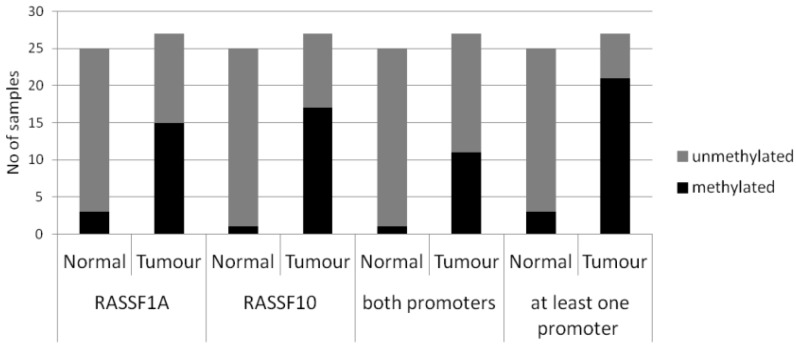
Promoter methylation summary of *RASSF1A* and *RASSF10* for single promoter methylation, both promoter methylation and number of samples that showed at least 1 methylated promoter. Statistically significant for all tumour *vs.* normal samples (Fisher Exact Test, two tailed).

We next studied primary breast cancer samples regarding a *RASSF10* and *RASSF1A* promoter methylation. A total of 27 breast tumour samples was analysed in comparison to 25 normal control tissues (Table 1 and Figure 3).

We found a promoter methylation of *RASSF10* in 17 out of 27 breast tumour samples (63%). However, only one out of 25 normal samples (4%) was methylated (*p* < 0.005). Additionally we analysed the methylation of *RASSF1A* and found promoter methylation in 15 out of 27 tumours (56%) but only in two out of 25 (8%) normal breast tissues (*p* < 0.005). In 40% of the breast tumour samples even both tumour suppressor promoters were methylated and almost 80% of tumour samples were carrying at least one promoter methylation (Figure 3). When studying the 13 matching breast tumour and control samples, the methylation degree was even more pronounced (Appendix Figure A1).

**Figure 4:**
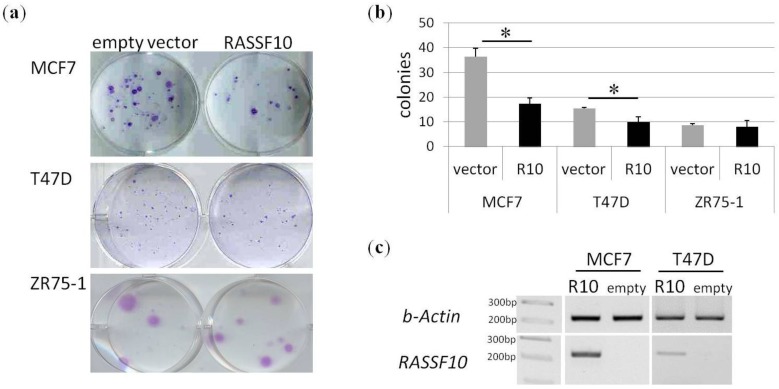
RASSF10 inhibits growth of breast cancer cell lines. (**a**) Colony formation analyses were performed in breast cancer cell lines MCF7, T47D and ZR75-1 after empty vector and RASSF10 overexpression. Cells were selected for three weeks using G418 and were Giemsa stained for visualization; (**b**) Quantification of colony formation in breast cancer cell lines MCF7 (*p* < 0.005) and T47D (*p* < 0.05) shows significant inhibition of colony growth under RASSF10. Statistically significant (asterisks) by paired T-Test, two tailed; (**c**) Overexpression verification of RASSF10 in MCF7 and T47D cell lines by RT-PCR.

Furthermore we tested the tumour suppressive function of RASSF10 in three breast cancer cell lines (Figure 4). MCF7 is a breast adenocarcinoma cell line, derived from metastatic site. T47D and ZR75-1 are cell lines from ductal carcinomas derived from metastatic sites (see ATCC for further details). The *RASSF10* status was methylated for MCF7 and T47D (Figure 2c). ZR75-1 was used as unmethylated negative control. RASSF10 expression plasmid and control plasmid were transfected in those cell lines and stably selected using G418 for three weeks. Subsequently colonies were stained with Giemsa (Figure 4a). Exogenous expression of RASSF10 inhibited colony formation in MCF7 and T47D, but not in the ZR75-1 control cell line (Figure 4a,b). RASSF10 overexpression (Figure 4c) significantly reduced number of forming colonies by half in MCF7 and by one third in T47D (Figure 4b).

**Figure 5:**
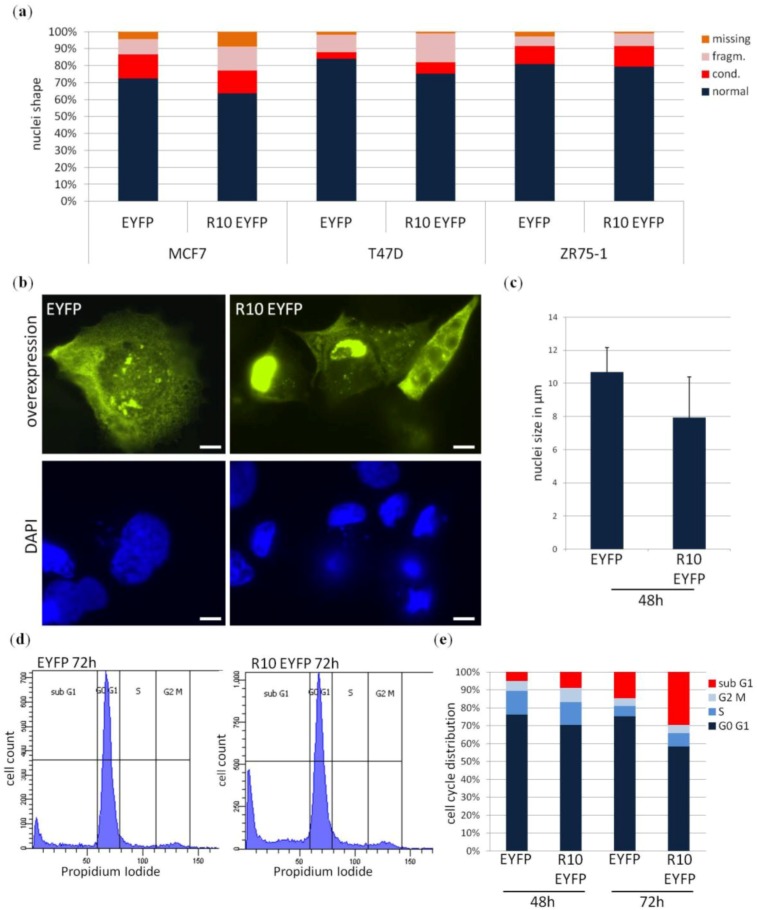
RASSF10 induces apoptosis in breast cancer cell lines. Overexpression of EYFP or RASSF10-EYFP in breast cancer cell lines induces morphological changes and DNA fragmentation associated with apoptosis induction. (**a**) Breast cancer cell lines MCF7, T47D and ZR75-1 exhibit alteration of nuclei shape after RASSF10 overexpression. Cells were analysed 72 h post transfection according to nuclei shape as normal, condensed, fragmented or missing (*n* = 100); (**b**) MCF7 cells show reduced nuclei size after RASSF10-EYFP transfections (5 μm standard) and (**c**) according nuclei measurements are shown (*n* = 17); (**d** and **e**) RASSF10 overexpression for 48 h or 72 h increases sub G1 fraction of transfected cells in flow cytometry analysis (*n* > 8000) using propidium iodide staining of DNA.

Next we aimed to understand how RASSF10 exerts its growth inhibition in breast cancer cell lines and performed apoptosis and cell cycle analysis. Apoptosis is typically associated with shrinkage of the cell and changes in nuclei morphology from condensation and fragmentation to karyorrhexis [26]. We found that overexpression of RASSF10 (tagged with EYFP) induced apoptotic nuclei in MCF7 and T47D (Figure 5a), both exhibit reduced RASSF10 expression due to promoter hypermethylation (Figure 2c).

In the breast cancer cell line ZR75-1, unmethylated at the RASSF10 promoter, overexpression of RASSF10 had no effect on nuclei morphology (Figure 5a). We observed that RASSF10 overexpression reduced not only the cell size of MCF7 cells but also the size of nuclei (Figure 5b). The according quantification showed that RASSF10 overexpression reduced the size of nuclei by more than 25% from 10.7 μm to 7.9 μm in diameter in comparison to EYFP alone (Figure 5c). We measured only nuclei with normal and condensed appearance, as an early sign of apoptosis, to determine nuclei shrinkage. To support our data of apoptosis induction by RASSF10 and to exclude an effect of RASSF10 reexpression on cell cycle distribution we performed flow cytometry analysis using propidium iodide staining (Figure 5d). Propidium iodide staining visualises the DNA content of cells. Cell cycle distribution can be measured in G1-G0-phase, S-phase, G2-M-phase and at the same time sub G1 fraction can be determined. RASSF10 overexpression had no influence on the cell cycle distribution as there are no alterations of cells in S-phase or G2-M-phase. However, RASSF10 reduced the number of cells in G0-G1 and increased the number of cells with a DNA content less than G1, an indicator of apoptosis with occurring DNA fragmentation. The tumour suppressive effect of RASSF10 was most prominent after 72 h when G0-G1 phase cell numbers were reduced from ca. 75% to 58%. In contrast, the number of cells in sub G1 increased from 15% to almost 30% in RASSF10 reexpressing MCF7 cells. In summary, we could show that RASSF10 induces apoptosis in breast cancer cell lines and therefore exhibits its growth inhibitory potential as a tumour suppressor.

## 3. Discussion

In our study we analysed the inactivating promoter methylation status of *RASSF10* and *RASSF1A* in breast cancer by COBRA and pyrosequencing analysis, as well as its implications in tumour growth by colony formation. We found that *RASSF10* is highly expressed in normal breast tissue and therefore studied breast tumour samples regarding a possible tumour driving *RASSF10* inactivation. *RASSF10* contains a more than 2 kb large CpG Island covering its promoter, and its methylation status was analysed by COBRA and pyrosequencing. Our own earlier work pinpointed *RASSF10* as a heavily inactivated tumour suppressor in various tumour types [16,20,21,22,23]. We were therefore interested in studying *RASSF10* in breast cancer and analysed normal *vs.* cancerous primary tissues. We found a significant *RASSF10* promoter methylation of 63% in tumour samples, compared to only 4% of methylation in the normal samples. Additionally we could show that *RASSF1A*, the most prominent member of the C-terminal RASSFs, exhibited 56% promoter methylation in breast cancer with only 8% methylation in normal breast tissues [9]. Interestingly, we observed that the vast majority of (80%) breast cancer samples showed methylation of one or both RASSF promoters, which implicates a growth advantage of breast cancer under RASSF promoter hypermethylation. However, we could not find a correlation with histological status due to the limited number of samples available. Interestingly, in our earlier findings in sarcoma we observed an increase in *RASSF10* methylation with tumour stage [16]. In primary thyroid tumours with affected lymph nodes *RASSF10* methylation was increased *vs.* those tumours from patients with unaffected lymph nodes [23]. In pheochromocytoma, cancer of the adrenal gland, we were able to study methylation all ten RASSF members. We observed a likewise tendency towards promoter inactivation of more than just one RASSF member in pheochromocytoma [20]. This indicates a common mechanism of inactivating several RASSFs that could drive tumour formation. This must likely be attributed to distinct functions of single RASSFs. Our present work additionally shows that inhibition of DNA methylation (by blocking DNMT action using Aza) in breast cancer cell lines reverses *RASSF10* promoter methylation. This demethylation was accompanied by reexpression of *RASSF10* in those breast cancer cell lines. Further reexpression of RASSF10 in two *RASSF10* methylated breast cancer cell lines blocked tumour growth in colony formation analysis. We also presented similar results in pancreas carcinoma and sarcoma tumours [16]. Our own earlier work implicated RASSF10 in inhibiting cell cycle progression in certain cancer cells upon activation of the cAMP-signalling pathway [16]. In MCF7 cells RASSF10 showed no effect on cell cycle progressions, but induced nuclei shrinkage and DNA fragmentation in breast cancer cells as signs of its tumour suppressor function. We therefore conclude that RASSF10 induced apoptosis in the *RASSF10* hypermethylated breast cancer cell lines MCF7 and T47D. Future studies will clarify the mechanisms of RASSF10 tumour inhibition in breast cancer in detail. Our present study clearly shows the tumour suppressive potential of the 10th family member of RASSFs and its strong promoter hypermethylation in primary breast tumours. We therefore concluded that *RASSF10* could serve as an indicator of breast tumour presence by analyzing DNA promoter methylation of *RASSF10* in e.g., biopsies or body fluids. Further larger samples numbers will clarify, if *RASSF10* methylation could be utilised as a prognostic tool in early breast cancer detection, predicting prognosis and susceptibility to chemotherapeutic response.

## 4. Materials and Methods

### 4.1. CpG Island Prediction, PCR Product Size and Digestion Products

The promoter region of *RASSF10* was analysed by CpG plot http://www.ebi.ac.uk/Tools/seqstats/emboss_cpgplot/ and shows the existence of a more than 2 kb large CpG island. Primers for bisulfite treated DNA were designed to bind only fully converted DNA and amplify promoter region RASSF10 (listed in Primers). The precise promoter region was chosen for CpG content and presence of according restriction enzymes for COBRA analysis. The *RASSF10* COBRA PCR product is 241 bp (with *Taq*I sites at 50 and 141). Summary of CpG island, COBRA PCR product, primer positions, *Taq*I restriction sites and pyrosequencing region are shown in Figure 1. *RASSF1A* promoter details were published earlier [9].

### 4.2. Breast Cancer Tissues and Controls

The analyzed primary breast tissues, including 27 tumour and 25 adjacent normal samples (Table A1), were characterised previously [9,27]. All patients signed informed consent at initial clinical investigation. The study was approved by COH ethic committee (City of Hope Medical Center, Duarte, CA, USA). RASSF1A promoter methylation was characterised previously [9].

### 4.3. DNA Isolation

Tissue specimens were deparaffinised by xylene and ethanol treatment. DNA was isolated with a QIAamp DNA extraction kit (Qiagen, Hilden, Germany) then proteinase K (Thermo Fisher Scientific, Dreieich, Germany) treated and concentrations of DNA were determined by UV-photospectrometery.

### 4.4. Methylation Analysis by COBRA and Pyrosequencing

2 μg genomic DNA from breast tissue or control tissue was bisulfite treated (12 μL 0.1 M hydroquinone, 208 μL 1.9 M sodium metabisulfite and pH 5.5 with NaOH) and incubated over night at 50 °C. DNA was purified using MSB Spin PCRapace (STRATEC Molecular, Berlin, Germany), eluted in 50 μL H_2_O and followed by 10 min incubation with 5 μL 3 M NaOH at 37 °C. DNA was then precipitated with 100% ethanol and 7.5 M ammonium acetate and resolved in 1x TE buffer. 200 ng were subsequently used for 25 μL PCR reaction with COBRA primers. The PCR product was digested with 0.5 μL of *Taq*I (Thermo Fisher Scientific) 1 h at 65 °C and resolved on 2% TBE gel together with mock control. Pyrosequencing was performed according to manufacturer’s protocol with PyroMark Q24 System (Qiagen). *In vitro* methylation of genomic DNA was performed using CpG Methyltransferase M.SssI (NEB) according to manufacturer’s protocol.

### 4.5. RNA Expression Analysis

RNA was isolated using Isol-RNA lysis procedure (5 Prime). RNA was DNase (Thermo Fisher Scientific) treated and then reversely transcribed by MMLV (Promega, Mannheim, Germany). Quantitative RT–PCR was performed in triplicate with SYBR select (Thermo Fisher Scientific) using Rotor-Gene 3000 (Qiagen, Hilden, Germany). Normal human RNAs were obtained from Agilent Technologies (Waldbronn, Germany). All other RNAs were isolated from cell culture.

### 4.6. Cell Culture, Aza Treatment and Colony Formation Analysis

Cell lines were grown in appropriate medium (DMEM or RPMI) supplemented with 10% FCS and 1% Penicillin/Streptomycin under cell culture conditions (37 °C, 5% CO_2_). For 5-Aza-2′deoxycytidine (Aza) treatment cells were split to 10% density and Aza was added with fresh medium on 4 consecutive days before RNA and DNA was isolated. Breast cancer cell lines MCF7, T47D and ZR75-1 were chosen for growth assay due to known *RASSF10* promoter methylation status. Cell lines were transfected using Turbofect (Thermo Fisher Scientific) or Nanofectin (PAA, Cölbe, Germany) and 4 μg of RASSF10 expression plasmid pCMVTag1 and mock transfected with empty pCMVTag1. The following day selection started with G418 (Biochrom, Berlin, Germany) for 3 weeks. Upon visible colony formation cells were dried and stained with Giemsa. Statistical analysis was performed using paired *T*-Test, two tailed.

### 4.7. Apoptosis Assays

Cell lines MCF7, T47D and ZR75-1 were seeded in 6 well plates on glass slides and transfected the following day with 4 μg of EYFP or RASSF10-EYFP using Turbofect (Thermo Fisher Scientific). 48 h or 72 h after transfection cells were fixed with formaldehyde, stained with DAPI (0.1 μg/mL in PBS, Sigma Aldrich, Taufkirchen, Germany) and embedded in Mowiol (Sigma Aldrich). Transfected cells (*n* > 100) were analysed for normal, condensed, fragmented or missing nucleus using Axio Observer Z1 (Zeiss, Jena, Germany). Volocity Software (Perkin Elmer, Baesweiler, Germany) was used for nuclei shrinkage measurements. For cell cycle/apoptosis analysis cells were grown on 10 cm plates and were transfected with 10 μg of EYFP or RASSF10-EYFP using Turbofect (Thermo Fisher Scientific). After 48 h and 72 h cells were isolated using Trypsin (Gibco, Thermo Fisher Scientific). Cells were fixed with ethanol over night at −20 °C. The following day cells were treated with 50 μg/mL RNase A for 30 min at 37 °C. For flow cytometry assay cells were stained with 50 μg/mL propidium iodide prior to measurement of DNA content in FACSCantoII (BD Biosciences, Heidelberg, Germany). FACSDiva Software (BD Biosciences) was used for measurement and gating to distinguish transfected fluorescence cells and to determine sub G1, G0-G1, S and G2-M phase of the cell cycle.

### 4.8. Plasmids

*RASSF10’s* coding sequence was amplified from genomic DNA and cloned into pCMVTag1 (Stratagene, Agilent, Waldbronn, Germany) and EYFP (Clontech, Takara Bio Saint-Germain-en-Laye, France) plasmids (as published earlier [16]). Plasmids were controlled by sequencing and expression analysis by RT-PCR, western blotting and immunofluorescence.

### 4.9. Primers

Primers for COBRA analysis of the RASSF10 promoter were upper primer ATAAGTAGAGGAGTTAGTAGGTTAAAGGAGA and lower primer AAATACAAAAAACTCAA AACCCAAACCC. For Pyrosequencing of *RASSF10* lower primer was biotinylated. For pyrosequencing of the *RASSF10* promoter the upper primer GTGGAGGGATTTTTGAATTTTTTTT was used. Primers for RT-PCR were *b-Actin* upper primer CCTTCCTTCCTGGGCATGGAGTC and lower primer CGGAGTACTTGCGCTCAGGAGGA and *RASSF10* upper primer GCGCCATGGAT CCTTCGGAAAA and lower primer GGCAGCGCCTCGTCGTCGTCCT.

## Figures and Tables

**Table 1:** Promoter methylation of *RASSF1A* and *RASSF10* in primary breast tumours.

Primary Breast Tissue	Cases	Promoter Methylation (%)				Statistical Test
		RASSF1A		RASSF10		
**Normal**	*n* = 25	2/25 (8%)	*p* < 0.005	1/25 (4%)	*p* < 0.005	Fishers Exact two tailed
**Tumours**	*n* = 27	15/27 (56%)		17/27 (63%)		

## Appendix

**Table A1:** RASSF methylation of primary breast tumours.

No.	Type	Details Histology	Grade	Promoter Methylation	
				RASSF1A	RASSF10
B1	Tumor	infl. poor diff ductal BrCA	3	m	m
B2	Tumor	infl. poor diff ductal BrCA	n.a.	u	u
B3	Tumor	infl. ductal BrCA	3	m	u
B4T	Tumor	infl. ductal BrCA	2-3	u	m
B4N	Normal			u	u
B5	Tumor	infl. ductal BrCA	3	u	u
B6	Tumor	infl. poor diff ductal BrCA	3	m	m
B7T	Tumor	breast cancer	n.a.	m	m
B7N	Normal			u	u
B8T	Tumor	BrCA	n.a.	m	m
B9T	Tumor	ductal AdenoCA	n.a.	u	m
B9N	Normal			u	u
B10T	Tumor	well diff. infl. ductal AdenoCA	1	m	u
B10N	Normal			u	u
B12T	Tumor	infl. ductal BrCA	3	m	m
B12N	Normal			u	u
B28T	Tumor	infl. moderatly diff ductal CA	2	u	m
B28N	Normal			u	u
B33T	Tumor	colloid BrCA	n.a.	m	m
B33N	Normal			u	u
B34T	Tumor	invasive BrCA	3	m	m
B34N	Normal			u	u
B36T	Tumor	infl. moderatly diff ductal CA	2	m	m
B36N	Normal			m	m
B38T	Tumor	infl. moderatly diff ductal CA	2	m	m
B38N	Normal			u	u
B47T	Tumor	infl. ductal BrCA	3	m	u
B47N	Normal			u	u
B26T	Tumor	invasive mammary ductal	n.a.	m	m
B26N	Normal			u	u
B27T	Tumor	invasive mammary ductal	n.a.	m	u
B27N	Normal			u	u
B20N	Normal			m	u
B23N	Normal			u	u
B29N	Normal			u	u
B31N	Normal			u	u
B40N	Normal			u	u
B41N	Normal			u	u
B43N	Normal			u	u
B44N	Normal			u	u
B45N	Normal			u	u
B46N	Normal			u	u
B50N	Normal			m	u
B51N	Normal			u	u
B15T	Tumor	infl. ductal BrCA	3	u	u
B18T	Tumor	infl. lobular BrCA	2	u	u
B21T	Tumor	ductal AdenoCA	3	u	u
B24T	Tumor	invasive mod. diff. ductal AdenoCA	2	u	u
B30T	Tumor	infl. mod.diff. ductal AdenoCA	2	u	m
B32T	Tumor	infl. ductal carcinoma	3	m	m
B37T	Tumor	infil. ductal AdenoCA	3	u	m
B39T	Tumor	infil. ductal AdenoCA	2	u	m

Abbreviations: CA carcinoma, BrCA breast carcinoma, n.a. not available, m methylated, um unmethylated, T Tumour, N Normal. Matching samples carry with same number.
